# Additive Manufacturing Provides Infinite Possibilities for Self‐Sensing Technology

**DOI:** 10.1002/advs.202400816

**Published:** 2024-05-20

**Authors:** Daobing Chen, Zhiwu Han, Junqiu Zhang, Longjian Xue, Sheng Liu

**Affiliations:** ^1^ The Institute of Technological Science Wuhan University South Donghu Road 8 Wuhan 430072 China; ^2^ The Key Laboratory of Bionic Engineering (Ministry of Education) Jilin University Changchun Jilin 130022 China; ^3^ School of Power and Mechanical Engineering Wuhan University South Donghu Road 8 Wuhan 430072 China

**Keywords:** additive manufacturing, intelligent components, proprioceptors, self‐sensing

## Abstract

Integrating sensors and other functional parts in one device can enable a new generation of integrated intelligent devices that can perform self‐sensing and monitoring autonomously. Applications include buildings that detect and repair damage, robots that monitor conditions and perform real‐time correction and reconstruction, aircraft capable of real‐time perception of the internal and external environment, and medical devices and prosthetics with a realistic sense of touch. Although integrating sensors and other functional parts into self‐sensing intelligent devices has become increasingly common, additive manufacturing has only been marginally explored. This review focuses on additive manufacturing integrated design, printing equipment, and printable materials and stuctures. The importance of the material, structure, and function of integrated manufacturing are highlighted. The study summarizes current challenges to be addressed and provides suggestions for future development directions.

## Graphical abstract

Proprioception embedded in biological bodies can be migrated to smart devices, depending on the right configuration of materials and structures in the right place. Additive manufacturing technology can meet this very well, which provides unlimited possibilities for the preparation of self‐sensing intelligent devices.

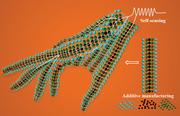

## 1 Introduction

**Figure 1:**
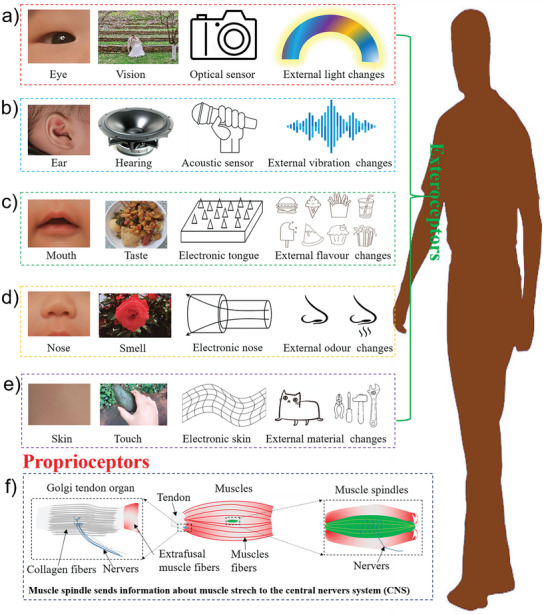
The exteroceptors and proprioceptors of the human body and the types of sensory signals and corresponding artificial sensors. a) The eyes can sense changes in external light and form visual perception, and the corresponding human invented a variety of optical sensors; b) ears help us receive speech information and form hearing by sensing vibration. Correspondingly, people have invented a variety of acoustic sensors; c) people sense the taste of food through the tongue in the mouth and invent the corresponding electronic tongue; d) the nose allows people to sense smells in the environment and distinguish dangers, so the electronic nose was invented accordingly; e) people's skin can feel temperature, material, water and so on through touch. Accordingly, people have invented a variety of flexible electronic skin sensors; f) the proprioceptor of human body can monitor the proprioceptive activity of muscles in real time, including muscle stretch, health, and growth. Accordingly, a variety of self‐sensing devices have been invented.

Sensors are devices that obtain measurements and convert them into electrical signals or other information according to specific rules to enable information transmission, processing, storage, display, recording, and control.^[^ ^1^, ^2^, ^3^, ^4^ ^]^ They are core components of intelligent devices and can perceive touch,^[^ ^1^, ^5^ ^]^ taste,^[^ ^6^, ^7^, ^8^ ^]^ vision,^[^ ^9^, ^10^ ^]^ hearing,^[^ ^11^, ^12^ ^]^ and smell,^[^ ^13^, ^14^ ^]^ similar to human senses (**Figure** **1**a–e).^[^ ^15^ ^]^ However, research on intelligent equipment has focused on external sensors^[^ ^16^, ^17^ ^]^ to obtain more accurate information but less on internal sensing.^[^ ^18^, ^19^ ^]^ The absence of internal information can potentially lead to inaccurate data and inadequate self‐evaluation, ultimately culminating in significant failures. Organisms have developed external receptors and high‐performance internal proprioceptors (Figure 1f).^[^ ^16^, ^17^, ^18^, ^19^ ^]^ External receptors are capable of detecting changes in the external environment in real time, whereas proprioceptors can assess the organism's condition in real‐time. The seamless integration of both external and internal receptors is essential for an organism to achieve lightness, agility, and vitality. Therefore, it is necessary to equip intelligent devices with high‐performance external and internal ontology sensors.^[^ ^20^, ^21^, ^22^ ^]^ The self‐sensing capabilities of intelligent devices have garnered significant attention in the fields of sensors and engineering, making it a highly discussed topic.^[^ ^23^, ^24^, ^25^, ^26^, ^27^, ^28^, ^29^ ^]^

The most important characteristic of self‐sensing technology (SST) is the ability of the device to assess the stability of its structure and components.^[^ ^16^, ^17^, ^18^, ^19^ ^]^ This capability has a wide range of applications in construction, biomedical, aerospace, military, microelectronics, and other civilian fields.^[^ ^23^, ^24^, ^25^, ^26^, ^27^, ^28^, ^29^ ^]^ In the early stages of self‐sensing research, the focus was primarily on the exploration and development of self‐sensing cement and its application in diverse structures, encompassing bridges, tunnels, skyscrapers, residential buildings, highways, and water conservancy facilities.^[^ ^30^ ^]^ Self‐sensing cement can detect stress and strain and assess building health to prevent damage and loss of life and property. The application of self‐sensing devices has broadened in recent years, and these devices have been used as key components of intelligent equipment.^[^ ^29^, ^31^, ^32^, ^33^ ^]^ For instance, an intelligent robot armed with a self‐sensing hand is capable of perceiving deformation. Within the biomedical realm, implantable prostheses constructed with self‐sensing materials can generate a magnetic field. In the event of implant damage, the magnetic field undergoes alterations.

A self‐sensing intelligent device (SSID) includes self‐sensing actuators and other devices.^[^ ^30^, ^32^, ^33^, ^34^, ^35^ ^]^ The SSID can detect changes in mechanical, chemical, magnetic, and optical signals. The majority of research on SSID has been centered on alterations in mechanical signals, encompassing a wide range of factors such as strain, stress, pressure, hardness, toughness, shear force, displacement, wind load, fracture, and failure.^[^ ^30^, ^32^, ^33^, ^34^ ^]^ Research on magnetic signals includes magnetostriction and self‐sensing of the magnetic field signal.^[^ ^35^, ^36^, ^37^ ^]^ Only a few self‐sensing studies investigated changes in chemical or optical properties. Chemical signals that can be detected by these devices include glucose, toxins, and harmful gases.^[^ ^38^, ^39^, ^40^, ^41^ ^]^ Detectable optical signals include transparency.^[^ ^29^ ^]^ While there have been limited studies exploring the self‐sensing capabilities of chemical and optical signals, these research domains are anticipated to become the focal points of future SST advancements.

The manufacturing process of SSIDs is contingent upon the characteristics of the materials utilized, with various materials being well‐suited to distinct processing techniques, encompassing both traditional and specialized methods. Most methods include chemical synthesis, pressing, casting, molding, mechanical processing, and other traditional processing methods.^[^ ^32^, ^42^, ^43^, ^44^, ^45^, ^46^, ^47^ ^]^ Only a few additive manufacturing (AM) processing methods exist.^[^ ^27^, ^48^, ^49^, ^50^ ^]^ Functional materials are commonly used for SSIDs, including conductive polymer composites, piezoelectric materials, magnetic polymer composites, photoresponsive materials, and other materials.^[^ ^35^, ^36^, ^37^, ^51^, ^52^, ^53^, ^54^, ^55^, ^56^, ^57^, ^58^ ^]^ These materials can convert external stimuli into electrical, magnetic, and optical signals. Together, they have high strength and stiffness, are used as actuators, and can be used as structural materials. For instance, the material employed for self‐sensing actuator devices often comprises a conductive shape memory polymer composite. Tailored processing techniques are utilized to guarantee that the device's resistance varies in tandem with alterations in its shape. These examples indicate that functional materials are critical components of SSIDs. However, structural design of SSIDs is required to ensure suitable material properties and characteristics. An appropriate structure and material often provide better performance and functionality. Self‐sensing, intelligent components often have complex, multi‐scale structures. They include various forward design and bionic structures. The forward design structure includes a pyramid, negative Poisson's ratio, lattice, and three‐cycle minimal surface structures.^[^ ^27^, ^53^, ^59^ ^]^ The bionic structure includes hexagonal, serpentine, slit, hair, and comb structures.^[^ ^33^, ^60^, ^61^, ^62^, ^63^ ^]^ They can be millimeter, micron, nanometer, nano‐to‐micron, micron‐to‐millimeter structures. It is challenging to prepare suitable materials and structures for SSIDs. Growth is a fundamental characteristic of living organisms, involving the sequential accumulation of material through physical and chemical processes. This progression ranges from inorganic to organic substances, from small molecules to macromolecules, from macromolecules to organelles, and ultimately from organelles to cells. Various materials are used to create different structures with different functions. AM mimics this natural process. It simulates growth to create various industrial products.^[^ ^64^, ^65^, ^66^, ^67^, ^68^ ^]^ It can be used to create SSIDs with various self‐sensing functions using different materials and structures.

This paper reviews research on additive manufacturing of self‐sensing intelligent devices (AMSSIDs) in recent years and examines the problems and challenges faced in this field. First, SST and SSID are briefly introduced, including their concepts, advantages, and disadvantages. Next, the development status of AMSSIDs is described, including the design, equipment, and materials. Finally, we examine the future prospects of AMSSIDs.

## 2 The Self‐Sensing Concept

The concept of self‐sensing is based on the proprioceptors of organisms (Figure 1f).^[^ ^16^, ^17^, ^18^, ^19^ ^]^ Proprioception, the human sense that enables us to perceive the movement of body parts, is responsible for involuntarily controlling the trunk and limbs.^[^ ^19^ ^]^ It is achieved by the nervous system through the coordination between the tendon spindle, muscle spindle, ligament, and other skin senses and the adjustment of local joint and muscle tension (Figure 1f). If proprioception is reduced, the nervous system has less control, and the chance of injury is greatly increased. The functions and concepts of ontology sensors in engineering applications are based on proprioception to enable the self‐sensing of devices.^[^ ^23^, ^24^, ^25^, ^26^, ^27^, ^28^, ^29^ ^]^ Self‐sensing devices are functional apparatuses that possess the ability to detect changes in their physical or chemical signals, including shape, pressure, temperature, and composition.^[^ ^23^, ^24^, ^25^, ^27^, ^28^, ^29^ ^]^ Most self‐sensing devices are transducers because they perform signal transmission and conversion, not signal analysis and processing. In general, the signals converted by the self‐sensing component are electrical signals, including resistance, voltage, current, and capacitance signals. They can also be optical signals, such as color changes.^[^ ^30^, ^32^, ^33^, ^34^, ^35^ ^]^

**Figure 2:**
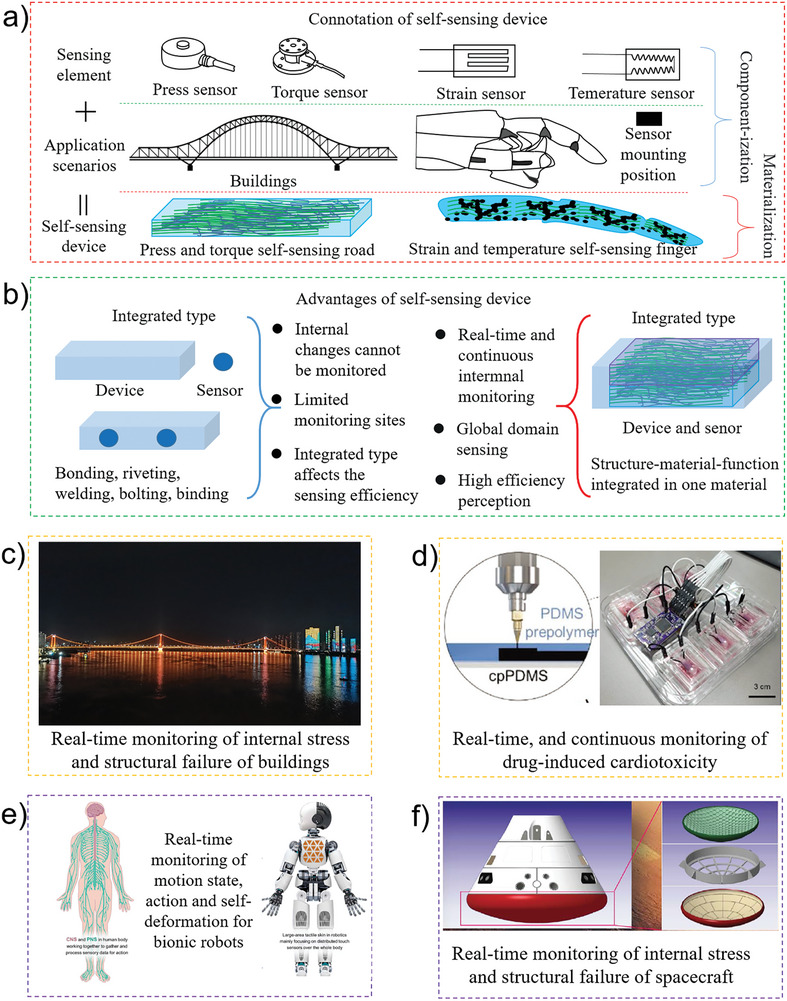
The connotation, advantages, and application scenarios of self‐sensing techonology. a) The transformation of traditional devices into intelligent devices requires the integration of sensors in the way of components, while self‐sensing intelligent devices (SSID) integrate sensors and devices in a materialized way and integrated in the process of fabrication; b) SSID can monitor the internal situation of the device in real time, and can realize global monitoring instead of point monitoring. Since the sensor itself is also a body part of the device, it can also greatly improve the sensing efficiency; c) SSID can be widely used to monitor the internal stress and structural failure of buildings, such as Bridges, tunnels, buildings, water conservancy facilities, etc; d) SSID can be used for the monitoring of own information of wearable medical devices, medical robots, and implantable medical devices, such as the monitoring of drug toxicity. Reprinted with permission.^[^ ^38^ ^]^ Copyright 2023. Wiley; e) SSID is needed to monitor the motion state, action, and deformation of robots, especially bionic robots, and flexible robots. Reproduced with permission^[^ ^73^ ^]^ Copyright 2022. AAAS; f) composite functional materials with self‐sensing technology can be widely used in aircraft, spacecraft, and space station. Reproduced with permission^[^ ^74^ ^]^ Copyright 2021. AAAS.

SSIDs stand apart from other functional components due to their intricate composition of complex integrated components and materials. Notably, the self‐sensing functional component exhibits both sensing and structural functionalities (**Figure** **2**a). For example, real‐time monitoring of bridge stress is required for bridge safety. The traditional method consists of placing multiple stress and torsion sensors in dangerous stress areas of the bridge. The less dangerous areas are rarely or never monitored. Moreover, this method can only monitor stress on the surface. More advanced methods or ultrasonic instruments are required to assess internal stress, which is time‐consuming and laborious and does not assess all parts of the structure. Using self‐sensing concrete to build bridges can solve this problem. The self‐sensing concrete material is mixed with carbon fiber or other conductive materials. The resistance remains uniform throughout the entire structure. However, whenever the concrete material encounters stress or experiences an internal fracture, the resistance alters, thereby facilitating real‐time monitoring of internal stress and fractures. Another example is the self‐sensing function of a mechanical finger. The stress and temperature of traditional manipulators are measured using strain gauges and temperature sensors, with the disadvantages of regional limitations and internal monitoring. An appropriate material configuration and structural design can transform a mechanical finger into a self‐sensing component that can detect finger activity, strain, and temperature in real time.

The integration of a self‐sensing device, a sensor, and functions enables the intelligent detection of various functions. The mechanical finger comprises self‐sensing material, facilitating the seamless integration of single, composite, or multiple materials. The fabricated component comprises support and sensing components that enable grasping. Separate integration methods are used to integrate the sensor and the functional components, including binding, bolting, riveting, and bonding. Some interfaces are unstable, and the components may become detached. The sensor only performs sensing, and the structure has a support function.

Self‐sensing functional components have the following advantages (Figure 2b): 1) the sensors are part of the component so that they can accurately sense the physical or chemical parameter changes in each part. 2) The self‐sensing functional component is an integrated device; thus, no additional sensor integration, packaging, and other processes are required, simplifying the manufacturing process. 3) Sensor integration enhances sensing efficiency while minimizing volume requirements, enabling the sensor to accurately determine its state. SSIDs have applications in many fields because of these advantages. They were first applied in the construction industry, with a research focus on self‐sensing cement composite materials.^[^ ^69^, ^70^ ^]^ Concrete or brick prepared with self‐sensing cement can be used to monitor the structural health of buildings, corrosion, and damage or fractures (Figure 2c).^[^ ^71^ ^]^ Self‐sensing components have been used in various application scenarios, including biomedicine, humanoid robots, aerospace, intelligent fabrics, and flexible electronics.^[^ ^33^, ^72^ ^]^ Since SST is based on proprioception, it has been used extensively in medical applications (Figure 2d).^[^ ^38^ ^]^ Self‐sensing flexible sensors are widely used for early disease detection. They enable the continuous monitoring of human physiological signals (body temperature, heart rate, muscle movement, etc.) and detect and prevent related diseases, such as heart failure, sleep apnea, Parkinson's disease, stroke, cardiovascular disease, and hypertension. The exceptional comfort, portability, flexibility, real‐time data acquisition, and robust security of self‐sensing flexible sensors promise to revolutionize early disease detection. Self‐sensing devices are also critical in the treatment stage. For example, a fixed‐point quantitative release of drugs in the human body can substantially improve the patient's health and reduce unnecessary cell damage. Smart micro‐ or nano‐scale balls are used for this purpose. The sensors monitor when and where the drug has been released. SSIDs are also used in the rehabilitation stage for real‐time, efficient, and continuous monitoring of physiological signals. Another major application of SSIDs is intelligent robotics, especially humanoid robotics (Figure 2e).^[^ ^73^ ^]^ The two core components of the robot are the actuator and the sensor. SSIDs comprise the sensor and actuator, i.e., a self‐sensing actuator, which improves the monitoring range and sensing efficiency. Self‐sensing actuators include magnetic, electric, pneumatic, and shape memory material actuators. For example, Xie et al.^[^ ^35^ ^]^ proposed a novel self‐sensing, large magnetostrictive actuator that used the Delta E effect to sense the actuator's stiffness. The experimental results show that the proposed SSID achieves self‐sensing output displacement within a stroke of nearly 50 µm, the sensitivity is 2.49 mV µm^−1^, and the self‐sensing displacement resolution can reach 63.4 nm. This innovative self‐sensing actuator, boasting micron‐scale self‐sensing actuation capabilities, can be seamlessly integrated into external sensorless execution systems. Self‐sensing intelligent components will be extensively used in future aerospace applications (Figure 2f).^[^ ^74^ ^]^ Aerospace equipment is operated at high altitudes, in deep space, and on exoplanets, often facing extremely harsh environments, such as alternating large loads, high and low temperatures, and strong radiation. Therefore, this equipment must be highly reliable. The safety and reliability of aerospace equipment can be significantly improved if the load, fatigue, internal damage, and material degradation can be continuously monitored in real time. For example, self‐sensing composite materials can be used for the aircraft skin to perceive changes in temperature, aerodynamic load, and gas flow velocity, enabling the real‐time adjustment of the flight attitude to reduce air turbulence and improve safety and comfort.

In summary, SST is inspired by proprioception and has been more widely used in engineering practice and applications. Similar to the ability of the proprioceptive muscle spindle and tendon spindle to monitor muscle activity, SSIDs can perform self‐sensing behavior, or the self‐sensing material can combined with ordinary materials. SSIDs can monitor mechanical, chemical, optical, and magnetic signals. SSIDs have the advantages of real‐time, continuous, global, and efficient perception. SST is widely used in construction, biomedical, intelligent robots, aerospace, and other fields; this sensing technology has expanded from external to internal applications.

## 3 Additive Manufacturing of Self‐Sensing Devices

Self‐sensing intelligent components are comprised of numerous materials, exhibiting multiple scales and intricate structures. The sensing materials are integrated into the interior of the device and act as structural support or driving materials. Selecting the apt location, structure, and integrating sensing materials with other materials poses a significant challenge in the production of self‐sensing intelligent components. Traditional material processing and manufacturing methods often do not meet the harsh requirements, whereas AM does.^[^ ^75^, ^76^, ^77^, ^78^, ^79^, ^80^ ^]^ AM manufacturing uses material deposition to build devices, in which the right material can be combined into the right structure and size at the right location. **Table** **1**. statistics the latest AMSSID research and shows the great application potential of AM in SSID preparation.^[^ ^75^, ^76^, ^77^, ^78^, ^79^, ^80^, ^81^, ^82^, ^83^ ^]^ It can be seen from Table 1 that currently, for AM preparation of SSID, various printing processes can be used, and these processes include FFF, FDM, DIW, DLP, 2PP, etc. More importantly, AMSSID can realize the printing of a variety of shape structures, including honeycomb, long strip, multilayer structure, cavity, core‐shell structure, lattice structure, cell unit structure, etc. The arbitrary design of the structure helps the SSID to achieve more excellent functions and performance. Although AM has unparalleled advantages in manufacturing SSIDs, current methods require improvement and long‐term development. The development process of AMSSIDs includes device design, material development, processing equipment, and performance evaluation. Based on the current development status and trend of AMSSIDs, we discuss four aspects: design, processing equipment, and material development.

**Table 1:** The latest results of AMSSID in recent years.

Ref.	Materials	Printing technology	Stucture	Self‐sensing fuctions	Sensing signal	Application prospect	Year
[75]	PLA/TPU continuous carbon fiber	FFF	Honeycomb	Stain Temperature	piezoresistivity	structural health monitoring	2022
[52]	Multiwall carbon nanotube (MWCNT)/polypropylene random copolymer	FFF	Strip	Stain Temperature	thermo‐resistive	self‐sensing temperature and strain/damage state	2021
[53]	multi‐walled carbon nanotubes (MWCNTs)/ high‐density polyethylene (HDPE)	FFF	S‐shaped, Chiral, and Re‐entrant	Stain	piezoresistive	Self‐sensing smart materials and structures with tunable sensitivity.	2022
[28]	Cr20Ni80 fiber @PEEK filament, PEEK filament	FDM	Hand shape, Shutters	Stain, deformation	Resistance	Deformation self‐sensing monitor	2022
[49]	Shape Memory Polyurethane, Graphene PLA	FFF	multilayered structure	Stain	Resistance	crawling robot, deployable kayak, development of sports equipment	2020
[50]	Graphene polylactic acid, paper	FDM	bilayer structure	Stain	Resistance	Intelligent actuators	2021
[76]	Silico‐aluminate/alkaline solution/fly ash	DIW	Strip	Temperature	Resistance	Concrete structures	2020
[77]	Flexible TPU, conductive PLA	FFF	Gripper	Stain	Resistance	Pneumatic actuators	2020
[78]	dimethylaminoethyl methacrylate	2PP	3D optical microresonator	Biochemical variations in aqueous environment	Spectral shift	optical sensing platform	2020
[79]	Carbon nanotube (CNT)‐reinforced polypropylene random copolymer (PPR) nanocomposites	FFF	Strip, auxetic re‐entrant, and S‐unit cell Lattices	Stain	Piezoresistive	biomedical engineering, orthopedic braces	2022
[59]	Multiwall carbon nanotube (MWCNT) incorporated polypropylene random (PPR) copolymer	FFF	Gyroid, Kelvin, BCC PLate	Strain	Resistance	Patients‐specific biomedical devices	2022
[80]	Liquid crystal elastomer LCE ink, liquid metal (LM) ink	core–shell nozzles DIW	core–shell structure	Strain	Resistance	Intelligent soft robotics, reconfigurable soft electronics, and RF devices.	2021
[81]	Continuous carbon fiber filament, PA6 filament	FDM	Strip	Strain	Resistance	Structural health monitoring	2022
[82]	Polylactic acid/continuous carbon fiber	FFF	Strip	Strain	Resistance	Aerospace, robotics	2022
[83]	Continuous glass fibers /polyacrylonitrile (PAN)‐based continuous carbon fibers/ Polylactic acid (PLA)	Self‐developed FDM	Strip	Strain	Resistance	Smart prosthetic socket	2021
[29]	Liquid crystal elastomers	DLP	Strip	Deformation	Optomechanical self‐sensing	Soft robotics	2021
[27]	PZT/ resins	Self‐developed AM equipment	3D lattice	Stain	Piezoelectricity	Proprioceptive microrobots	2022
[51]	Ecoflex/ PDMS/ graphene	DIW	Strip, hand	Deformation	Capacitance	Soft robots	2023

### 3.1 Additive Manufacturing Integrated Design

**Figure 3:**
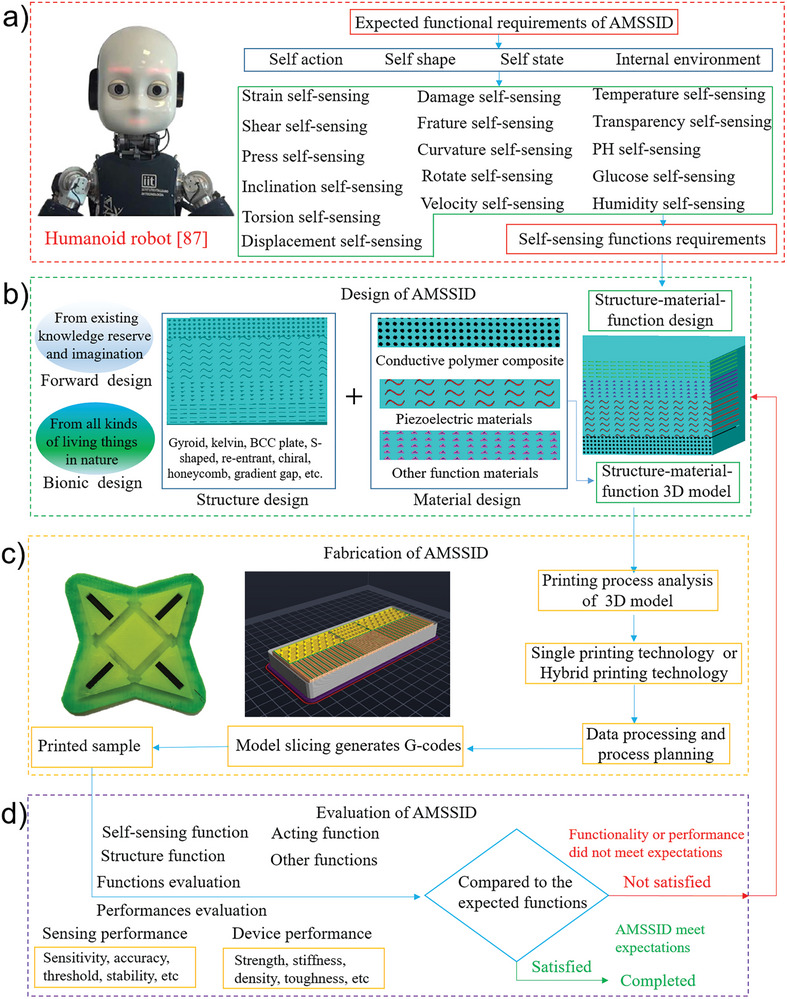
Additive manufacturing self‐sensing intelligent device (AMSSID) integrated printing manufacturing process. a) The function expectation of AMSSID mainly includes the self‐sensing function of its self action, self‐sensing function of its self‐shape, and self‐sensing of its own internal environment monitoring. For example, humanoid robots with multiple self‐sensing functions. Reproduced with permission^[^ ^87^ ^]^ Copyright 2021. AAAS; b) the integrated design of AMSSID mainly adopts the method of forward design and bionic design to put the right material and the right structure in the right position, so as to realize the expected self‐sensing function; c) the AMSSID model is processed and sliced, the printing path is planned, and the G code for printing is generated. Finally, input the printer for integrated printing and manufacturing; d) the AMSSID samples were evaluated, including the performance evaluation and functional evaluation of the samples. If the performance and functional requirements are met, the print is completed, if not, the print needs to be redesigned.

The design of AMSSIDs is similar to that of AM of functional components,^[^ ^84^, ^85^, ^86^ ^]^ but it has unique features. First, it is necessary to anticipate the functions of SSIDs. These functions are tailored to meet the specific requirements of the equipment, with indicators and performance criteria serving as the basis for evaluating their effectiveness. Reasonable design methods are adopted for the structural and material design, and a 3D model of the SSID is established, followed by simulations and optimizations. Then, appropriate AM equipment is selected or developed. The 3D printing process is constantly optimized to ensure that the printed sample of the SSID meets the design model. Finally, the function and performance evaluation of the SSID sample are carried out. If the requirements are met, the design and manufacturing of the SSID are completed; if it does not, the design scheme must be improved, or the printing process should be adjusted, and the processes should be repeated. The AMSSID is illustrated in **Figure** **3**.

It is imperative to analyze the anticipated functions of self‐sensing devices, as the SSID's functionalities are founded upon the specific requirements of the application. Typical methods are decomposition, aggregation, discretization, and coupling of the expected functions and the main indicators. The decomposition and analysis of the diverse functions necessitated by the self‐sensing device typically encompasses its primary, secondary, and inherent self‐sensing capabilities. The secondary and self‐sensing functions are also called auxiliary functions. Generally, the main function is the critical function of the self‐sensing device. Those of a humanoid robot^[^ ^87^ ^]^ include walking, gripping, chewing, swallowing, jetting, and jumping. A secondary function serves to enhance the performance or stability of the primary function. Typically, both the primary and secondary functions are defined by their ability to accomplish designated tasks, and they may interchangeably fulfill these roles. The index corresponding to the main function is the primary index, and that corresponding to the auxiliary function is the secondary index. When a humanoid robot executes its primary functions, such as walking, it must simultaneously perceive its own actions and form, while continuously monitoring internal changes in real‐time (Figure 3a). Monitoring signals include strain, shear, pressure, tilt angle, torsion, displacement, internal damage, fracture, temperature, transparency, pH, glucose content, and humidity.

Forward and reverse design methods are used in the AMSSID (Figure 3b). The forward design approach relies heavily on knowledge and experience, and is steered by the principles, techniques, and process models of system engineering. It is typically used in research and development and to improve the original design of SSIDs and the integration ability. Reverse design, which adopts a bionic approach, mimics the actions of animals and plants in order to design SSIDs utilizing additive manufacturing techniques. Both design methods are aimed at improving the self‐sensing component's functions. The most suitable design method is adopted to design a reasonable structure and material and choose a suitable position to obtain the expected self‐sensing function. Since the sensing function must be integrated with other functions, the characteristics of these functions must be considered when selecting materials. Single, composite, or multiple materials can be used. For example, the material utilized for a self‐sensing actuator must not only support the primary function but also exhibit conductivity, withstand specified forces, possess a suitable response speed, and demonstrate adequate strength. Often, a single material does not meet the requirements; thus, composite or multiple materials are used. After the material selection, the structure of the device is designed. A suitable structural design has appropriate properties. The sensing functions depend on the structural design, including forward design and bionic structures. For example, strain sensing requires grooves, filaments, or triangular, mastoid, sawtooth, corrugated, and Z‐shaped structures. Finally, a 3D model of the SSID is established to perform AM.

The next step is the AMSSID (Figure 3c). A comprehensive 3D model encompassing material, structural, and functional information is established, followed by simulations of the printing process and functionalities to ensure optimal model performance. Subsequently, the optimized multi‐information 3D model is sliced, data processing and process planning are performed, and the 3D printing equipment is selected. In the absence of commercial printing equipment, alternative suitable equipment is utilized. For example, Kotikian et al.^[^ ^80^ ^]^ fabricated a liquid crystal elastomer actuator with a self‐sensing function similar to nerves using coreless shell nozzle printing. A liquid crystal elastomer, liquid metal, and other materials were used. The liquid metal was encased in the liquid crystal elastomer. No commercial AM equipment exists for this material. Therefore, they developed a core‐shell printing device. The printing process must be continuously optimized to match the structure and material of the SSID to obtain high‐quality printed samples. The printing speed, filling method, extrusion speed, material viscosity, nozzle diameter, and substrate preheating temperature are optimized. In short, it is necessary to ensure the high quality of the printed sample so that it has high structural integrity.

Lastly, a thorough evaluation of the SSID sample's functionality and performance must be conducted. The evaluation criteria are determined by the intended function of the device. (Figure 3d). First, the function evaluation is conducted. SSIDs have multiple and coupled functions that require testing. For example, the functions of a self‐sensing actuator that necessitate testing encompass both sensing and execution capabilities, with the sensing function being of paramount importance. The sensing function accurately reflects the actuator states, including the shape, temperature, stress, and strain. A unified standard, detection technology, and detection equipment are lacking to perform functional evaluation of SSIDs created by AM. Given that various types of SSIDs necessitate distinct function evaluation criteria, it is imperative to assess and employ suitable methods and instruments tailored to each SSID type. Subsequently, the SSID performance is evaluated. High performance is required to execute the required functions. The performance evaluation encompasses a thorough assessment of the primary functions, as well as the sensing and self‐sensing capabilities of the device. The device performance is evaluated based on the strength, density, structural accuracy, and toughness. Sensitivity, accuracy, threshold, and stability are critical sensing performance criteria, and the self‐sensing performance is assessed by evaluating the synchronization, resolution, sensitivity, and accuracy. Finally, technical verification of the SST is conducted to establish a standardized and unified SSID record, encompassing technical details necessary for the standardized production of SSID products.

### 3.2 Printing Equipment and Technology

Self‐sensing functional devices often have special requirements of structure and materials. According to the characteristics of different self‐sensing components, different additive manufacturing equipment can be used. Firstly, for a single self‐sensing composite material, commercial single‐material or multi‐material additive manufacturing equipment can be used to complete it.^[^ ^39^, ^49^, ^76^, ^89^ ^]^ Of course, in the printing process, it is necessary to study the printing process according to the characteristics of the material in order to achieve better printing results. For example, in the process of DIW printing, the viscosity of the material has a great influence on the printing process.^[^ ^51^, ^76^ ^]^ The appropriate viscosity can make the material better extruded and will not collapse before curing. Then, for some complex SSID, it is necessary to make appropriate alterations to the existing equipment, including the transformation of the nozzle head, the transformation of the curing method, and the transformation of the combination of multiple printing processes.^[^ ^28^, ^80^, ^83^ ^]^ Finally, for SSID with special requirements or that cannot carry out additive manufacturing at present, it is necessary to develop a new type of additive manufacturing, so as to realize the printing of more intelligent materials and intelligent structures, and make the integration of materials and structures play the role of self‐perception.^[^ ^88^, ^95^ ^]^

Commercial or specialized equipment can be used for AM of SSIDs (typical application cases are listed in **Table** **2**), including fused filament fabrication (FFF), fused deposition modeling (FDM), direct‐ink writing (DIW), selective laser sintering (SLS), inkjet printing (Inkjet), stereolithography appearance (SLA), digital light processing, (DLP), and two‐photon polymerization (2PP). In addition, metal AM can also be used. We briefly introduce existing printing equipment for AMSSID and provide a future outlook.

**Table 2:** Different AM printing processes used for SSID preparation.

Printing technology	Type	Materials	Self‐sensing type	Superiority	Challenge	Ref.
Single	FDM/FFF	Thermoplastic material	Mechanical signal Temperature signal	Process and equipment simple and economical Good printing stability Easy to transform	Limited printable functional materials Surface line texture and low precision Multi‐material printing with defects material mixing	[49, 75, 81]
	DIW	Ink material (Volatile solvent, heat curing, light curing, sintering, soaking, etc)	Mechanical signal Temperature signal	Low equipment requirements Low manufacturing costs Wide range of raw materials Flexible manufacturing	Low printing accuracy Ink viscosity needs to be adjusted Many internal defects and collapse when printing flexible materials	[76, 80]
	Inkjet	Low viscosity high tension ink	Mechanical signal Temperature signal Chemical signal	High printing accuracy and good uniformity Easy to expand manufacturing	Material preparation difficult, process complex, and the required particularity High technical requirements and poor stability	[39, 92]
	SLS	Crystalline and semi‐crystalline polymers	Mechanical signal Temperature signal	Vast types printable materials Unsupported structure High material utilization rate and accuracy	Post‐processing difficult and easy to shrink High material loss Low printing efficiency	[89, 90, 91]
	SLA	Photosensitive resin material	Optical signal	Higher precision and higher printing efficiency Neat structure	Few material type Printing speed is slow and inefficient High cost of materials and equipment	[29]
	2PP	Photosensitive resin material	Optical signal	High printing accuracy Neat structure	Few material type High technical requirements and high cost	[78]
Hybrid	Inkjet&Screen print	Silver ink, piezoresistive ink	Mechanical signal	Multifunctional material integrated printing Across scales structures prepared separately Multifunctional areas are printed separately Complex structures integrated design and printing	Complicated process Hardware and software require special design The stability and fault tolerance of the printing process reducing Printing efficiency decreases and costs increase	[94]
	SLA&Inkjet	Photosensitive resin material, ink	Mechanical signal			[95]
	DIW& lectroplating	Metal, ink	Optical signal			[96]
	DIW&Inkjet	Conducting nanowires, extrusion ink	Mechanical signal			[88]

**Figure 4:**
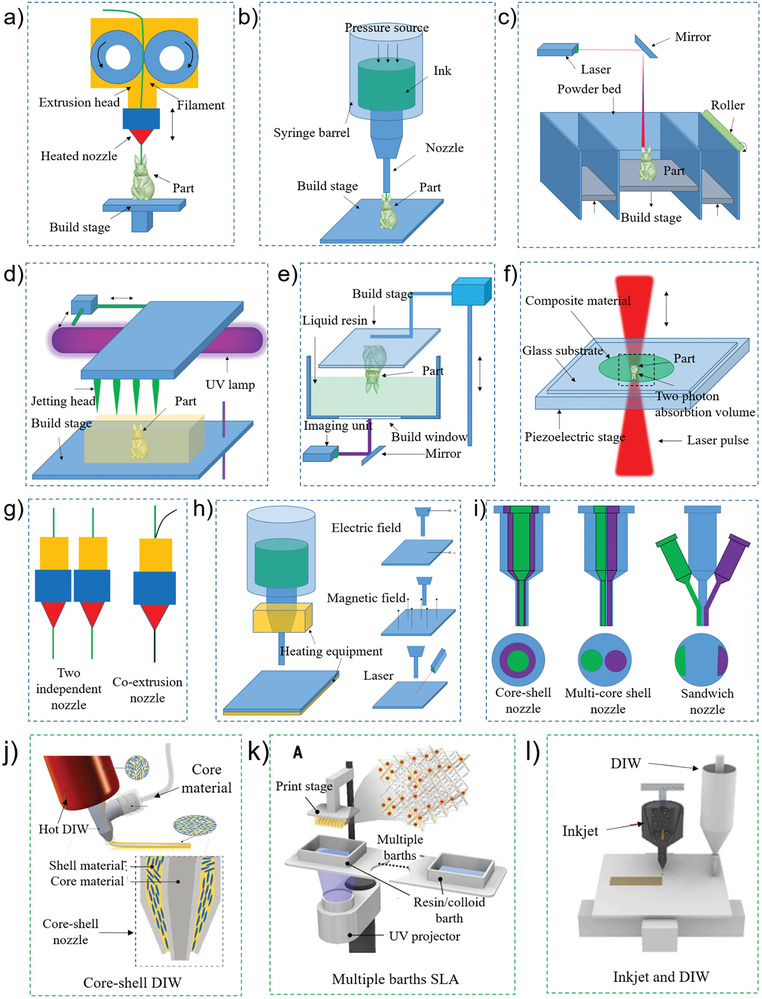
Additive manufacturing self‐sensing intelligent device (AMSSID) equipment, retrofitting equipment, and hybrid printing equipment. a) The most commonly used AMSSID manufacturing equipment is known as fused deposition molding (FDM) or fused filament fabrication (FFF). It is mainly used for SSID preparation of conductive polymer composites; b) direct‐ink writing (DIW) is a kind of AMSSID manufacturing equipment with high flexibility and modifiability; c) selective Laser Sintering (SLS) printing technology is also very suitable for the printing and preparation of AMSSID devices, since theoretically any powder material that can form inter‐atomic bonds after heating can be used as the molding material; d) inkjet printing technology is a kind of very potential microelectronic circuit printing equipment, and has a broad application prospect in AMSSID manufacturing; e) stereo lithography appearance (SLA) or digital light processing (DLP) has high printing accuracy, which can effectively improve the structural accuracy and device quality of AMSSID; f) two‐photon polymerization (2PP) printing technology can extend AMSSID samples to nanometer level accuracy; g) FDM can be used for multi‐material printing by adding print heads, or for AMSSID preparation by hybrid extrusion; h) DIW can be modified by adding heating equipment, external electric field, magnetic field, laser, solution immersion and other devices, so as to expand more printable materials and processes, and provide the possibility of printing multi‐material and complex structure AMSSID; i) extrusion printing equipment can transform the extrusion head, so as to realize the integrated printing of multiple materials and complex structures. The shape of the extrusion head can be core‐shell structure, multi‐core shell structure, or sandwich structure, and can also be a combination of a variety of other structures; j) self‐sensing liquid crystal elastomer device printed by modified core‐shell structure DIW printing equipment. Reproduced with permission^[^ ^80^ ^]^ Copyright 2021. Wiley; k) the SLA equipment is improved by adding different kinds of baths for the printing of self‐sensing piezoelectric functional components. Reproduced with permission^[^ ^27^ ^]^ Copyright 2022. AAAS; l) the hybrid additive manufacturing equipment was formed by combining INkjet and DIW and used for the preparation of AMSSID. Reproduced with permission^[^ ^88^ ^]^ Copyright 2021. Elsevier.

FFF/FDM is the earliest open‐source and widely used 3D printing (**Figure** **4**a). FFF/FDM materials are generally thermoplastic materials, such as wax, ABS, and nylon. The print material consists of a filament that is heated and melted in the extrusion head. While the extruded material is being dispensed, the extrusion head moves in the designated printing direction. The material quickly solidifies and bonds to the surrounding material. FFF/FDM printing technology has a simple principle, low maintenance cost, and safe system operation, making it highly suitable for the AMSSID. Most AMSSID uses this printing process, and a single or multiple materials can be used. For example, Ye et al.^[^ ^75^ ^]^ prepared polylactic acid/thermoplastic polyurethane (PLA/TPU) composites reinforced with continuous carbon fiber and FFF to fabricate self‐sensing components with a honeycomb structure. This self‐sensing component can sense the strain, temperature, and failure in real time.

Relatively few SSID samples have been created using multi‐material FFF/FDM, although this process will become more prevalent with further research.^[^ ^49^, ^50^, ^77^, ^81^ ^]^ For example, Iizuka et al.^[^ ^81^ ^]^ printed intelligent components capable of strain sensing using multi‐material printing and tested the components’ resistance during cyclic loading. Garces et al.^[^ ^49^ ^]^ printed a shape memory self‐sensing component with a multi‐layer structure using dual‐nozzle multi‐material fused thread manufacturing. The main materials were polyurethane with high shape memory and a graphene PLA composite. The latter was sandwiched between polyurethane layers. A strain change was achieved by changing the resistance. Hainsworth et al.^[^ ^77^ ^]^ produced a soft pneumatic mechanical gripper with a self‐sensing function using multi‐material FFF. The mechanical gripper comprised flexible TPU and conductive PLA, with the latter being printed alongside the TPU. When the flexible actuating gripper applies force, it's capable of detecting strain through resistance measurements.

Another common printing method used for AMSSID is DIW (Figure 4b). This technology was initially developed for the 3D manufacturing of ceramics and other materials. Subsequent research and development resulted in the current DIW method, which is widely used in microelectronic, photovoltaic, energy, tissue engineering, and other applications. A liquid or a solid–liquid mixture with high viscosity is used. The nozzle is installed on a three‐axis motion platform controlled by a computer. The nozzle is activated by mechanical or pneumatic pressure, and the ink is applied to the substrate. Several post‐treatments (volatile solvents, thermal curing, photocuring, sintering, and soaking) are applied depending on the material characteristics to obtain the 3D component. The primary advantage of DIW lies in its versatility in terms of applicable materials. This method can accommodate a wide range of materials, including composite materials, metals, polymers, ceramics, and hydrogels, thus rendering it ideal for the preparation of structurally complex and intricate samples, such as those required for SSID applications. For example, Vlachakis et al.^[^ ^76^ ^]^ printed cement components capable of temperature sensing using DIW. The main materials were silico‐aluminate, an alkaline solution, and fly ash. The printed self‐sensing cement component could sense high temperatures with an accuracy of 0.1 °C, and the long‐term sensing accuracy was 0.3 °C.

SLS is a rapid prototyping technology that uses an infrared laser as a heat source to sinter powder materials and create 3D parts using layer‐by‐layer stacking (Figure 4c).^[^ ^89^, ^90^, ^91^ ^]^ One of its key advantages lies in the ability to utilize a diverse range of materials. Theoretically, any powder material capable of being heated to form an interatomic bond can be employed in Selective Laser Sintering (SLS). As a result, this printing technology is exceptionally suitable for Advanced Manufacturing of Structurally Intricate Devices (AMSSID). For example, Ding et al.^[^ ^89^ ^]^ used an environmentally friendly method to prepare a mixed power of thermoplastic polyether block amide and multi‐walled carbon nanotubes for SLS, creating a conductive network structure of carbon nanotubes. The electrical properties, strain‐sensing capabilities, and shape memory performance of the flexible nanocomposites were thoroughly examined. SLS‐processed flexible sensors demonstrated regular, prompt, sensitive, and reliable cyclic responses during compression tests conducted with varying deformation strains and velocities. These findings suggest significant potential for their application in artificial electronic skin, man‐machine interfaces, and health monitoring systems. The nanocomposites had high shape recovery ability in the electrogenic shape memory cycle triggered by Joule heat at DC voltage. The prepared powder provides a new material for SLS and 3D printing. The strain‐sensing device exhibited excellent performance and multiple functions, making it suitable for developing flexible, lightweight, and wearable devices in the future.

Inkjet printing, a non‐contact printing technology, employs either thermal or piezoelectric mechanisms to dispense minute droplets of low‐viscosity ink onto a substrate. Subsequently, the ink undergoes polymerization upon exposure to an ultraviolet light source (Figure 4d). This technology is promising for microelectronic circuit printing and suitable for the manufacture of self‐sensing devices.^[^ ^39^, ^92^ ^]^ For example, Pu et al.^[^ ^39^ ^]^ proposed a continuous glucose detection technology based on flexible epidermal biological microfluidics. Utilizing inkjet printing micro‐manufacturing technology, they developed a wearable and flexible electrochemical glucose sensor patch equipped with a self‐calibration function. This innovative sensor demonstrated remarkable precision in dynamically and continuously monitoring glucose levels. The temperature control component and the glucose detection patch were fabricated separately. The former was prepared using inkjet printing by embedding a gold wire on the pretreated flexible polyimide film and heating it at 200 °C for 2 h. The self‐sensing temperature control module contained a flexible micro‐scale proportional–integral–derivative control circuit that shortened the measurement time and enabled continuous monitoring. The extraction of intercellular fluid occurred at a low current density, effectively eliminating the risk of allergic skin reactions. The module maintained the temperature of the detection device at 37 °C, ensuring optimal activity of glucose oxidase. Furthermore, inkjet printing was employed to fabricate the glucose detection patch, resulting in a flexible electrochemical glucose sensor seamlessly integrated with the extraction electrode pair. The measurement results were reliable due to the simple structure of the core device, and in situ measurements ensured high measurement inaccuracy. The flexible structure had close contact with the skin, avoiding measurement errors caused by the relative displacement between the sensor and the skin.

SLA/DLP (Figure 4e) and 2PP (Figure 4f) have been increasingly used in recent years. For example, Li et al.^[^ ^29^ ^]^ used DLP to prepare intelligent components that could sense the temperature based on the optical properties of materials. Due to the strong coupling between the shape response and the ordered network structure of a liquid crystal elastomer, its transparency undergoes significant changes as its shape is modulated by temperature variations ranging from room temperature to the phase conversion temperature. As the temperature increases, the material becomes more transparent, resulting in higher transmittance. The deformation of the material is measured by an optical signal, and it can be determined whether the deformation is due to manual or thermodynamical influences. In addition, the light intensity signal can be used to determine the bending degree, enabling sensing based solely on material properties. Saetchnikov et al.^[^ ^78^ ^]^ printed a micro‐resonant cavity using 2PP and measured the 4D spectral response. The device consisted of quencher‐doped sol‐gel SZ2080 material and can be used for detecting biochemical substances in water. The quality factor of the microresonant cavity was 4 × 104 at 685 nm. The 4D microcavity‐based sensor represents a novel device for optical sensing platforms with advanced properties suitable for a wide range of detection tasks.

The aforementioned SSID samples were initially prepared utilizing existing or commercially available equipment. Nevertheless, this machinery frequently falls short when it comes to fabricating SSID samples that involve multiple materials and possess a complex structural design. Consequently, there is a pressing need to modify the existing equipment to cater to these specific requirements. FDM equipment is modified by adding different print heads, performing co‐extrusion, and modifying nozzles (Figure 4g,i). For example, Zhou et al.^[^ ^28^ ^]^ selectively and sequentially heated a shape memory polymer composite to generate accurate and programmable deformation. A multi‐material FDM device was modified to create a sophisticated multi‐material printing apparatus, incorporating two extruders. One extruder is dedicated to extruding polymer monofilaments, while the other extruder is specifically designed for extruding polyetheretherketone and continuous metallic fiber. The continuous metallic fiber was surrounded by the molten polymer at the feed port and subjected to downward friction. In this way, the wire and support structure can be printed in an integrated way, so as to achieve better strain self‐sensing performance. In another example, Luan et al.^[^ ^83^ ^]^ used a multi‐material for in situ AM and prepared intelligent components with strain self‐sensing properties. The AM method encompassed five distinct printing directions and utilized two mixed printing nozzles. Nozzle 1 was responsible for depositing a continuous glass fiber‐PLA composite material, while nozzle 2 printed a continuous carbon fiber‐PLA composite material. This innovative setup allowed for the precise deposition of various composite materials, enhancing the functionality and performance of the final printed product. The composites exhibited excellent mechanical properties and could monitor the strain and damage of the component in real time.

Due to its straightforward structure and versatility in curing diverse materials, DIW is frequently employed in AM research for modifying the method and fabricating intricate structures incorporating multiple materials. This approach offers a flexible and efficient means to create complex objects with tailored properties and functionalities (Figure 4h,i). For example, Kotikian et al.^[^ ^80^ ^]^ developed a core‐shell printing nozzle to prepare a liquid crystal elastomeric actuator with a self‐sensing function similar to a nerve (Figure 4j). The liquid crystal elastomer was used as the shell, and liquid metal material was used as the core. The extruded core‐shell silk wire was activated by Joule heat and could sense length changes based on the resistance. Crucially, a closed‐loop control system was successfully implemented for the self‐sensing liquid crystal elastomer. This allows researchers to preset the desired resistance change, R/R0, and automatically regulate the resistance feedback throughout the deformation process, even amidst external disturbances. The target resistance change of the square waveform *R*/*R*_0_ = 0.90 and *R*/*R*_0_ = 0.65, and the corresponding strain change was ≈5% and 23%. Applying a load changed the actuator's resistance. The resistance changed significantly when the load was first applied. Subsequently, the resistance change was negligible. The maximum self‐adjusting load of the closed‐cycle control actuator was 4.2 g, which was 115 times the mass of the actuator.

The printing direction of piezoelectric materials is frequently altered in photocuring printing equipment. Piezoelectric materials, which exhibit the remarkable ability to transform electric fields into mechanical strains and vice versa, are highly suitable for endowing robotic systems with self‐sensing and actuating capabilities. However, due to the asymmetric displacement of the crystal ions, their electric‐induced strain is limited by the crystal structure. The manufacturing process involves extensive machining and assembly steps when piezoelectric materials are used as sensors in robotic systems, such as ceramic machining, lamination, connection of flat electrodes for activation and actuation, and integration with transmission mechanisms to amplify the piezoelectric strain and convert the strain into motion in the desired direction. Conventionally, these manufacturing methods primarily employ solid piezoelectric materials that often do not align well with the electrodes. This mismatch poses challenges in reducing the weight of the driving element and limiting the activation of the bidirectional piezoelectric effect to smaller scales. Cui et al.^[^ ^27^ ^]^ developed a charge‐programmed multi‐material AM technique to assemble conductive, piezoelectric, and structural materials into complex 3D microarchitectures to fabricate robotic metamaterials. The printing device included several replaceable material baths to cure the material under UV light (Figure 4k). The materials functioned as highly capable microrobots, performing a range of robotic tasks with ease. These tasks encompassed motion, steering, stepping, two‐way sound production, ultrasonic transduction, and decision‐making through self‐sensing feedback control, demonstrating their versatility and potential in various robotic applications. Numerical and experimental verification indicated that the 3D microstructure piezoelectric materials exhibited piezoelectric strain constants not obtainable with standard materials and produced electric‐field induced strain transformations, including tension, shear, torsion, and bending with different degrees of freedom and their combinations, as well as amplification of strain and logic‐dependent subtraction and addition of strain.

Another strategy to modify existing AM equipment is a hybrid printing process^[^ ^48^ ^]^ by combining traditional processing methods with AM^[^ ^48^, ^93^, ^94^ ^]^ or integrating different AM processes into one device. Examples include the combination of death printing and AM or casting, machining, and AM. For example, Verma et al.^[^ ^94^ ^]^ proposed a novel printing method for fabricating piezoresistive pressure sensors using aerosol jet printing (AJP) and screen printing. AJP is designed to print silver particles on SLS polyamide plates as custom substrates. Piezoresistive electrodes are manually screen‐printed on top of the interconnects as sensing layers. The sensor was electromechanically tested and its sensitivity, hysteresis, reproducibility, and time drift were evaluated. When slope pressure was applied, the sensor exhibited two sensitive zones: 1) a high‐sensitivity zone in the range of 0–0.12 MPa with an average sensitivity of 106 ω MPa^−1^, and 2) an average‐sensitivity zone with 7.6 ω MPa^−1^ in the range of 0.12–1.25 MPa; some overlapping regions were observed.

The most common mixing printing methods include FDM/DIW, DIW/DLP, Inkjet/SLA,^[^ ^97^ ^]^ and Inkjet/DIW.^[^ ^88^ ^]^ Jeong et al.^[^ ^95^ ^]^ proposed an SLA/Inkjet method and prepared the electromagnetic pressure sensors with metamaterial. An electromagnetic pressure sensor was meticulously crafted using flexible resin as its deformable substrate, with inkjet printing patterns applied on its surface. The core component of this sensor, the metamaterial absorber, was designed in the shape of a square ring. Given that the absorption frequency is intricately linked to the thickness of the substrate, this device can effectively function as an electromagnetic pressure sensor, leveraging its mechanically deformable substrate for precise measurements. The results showed that the absorption frequency increased from 5.2 to 5.66 GHz when 0 and 20 N of pressure was applied. The device sensitivity was 7.75 × 10^8^ Hz/mm. It could be reused 100 times. Du et al.^[^ ^88^ ^]^ demonstrated a piezoelectric device based on tellurium nanowires fabricated with a hybrid printing method (AJP and extrusion printing) on a single printing platform (Figure 4l). Owing to the distinctive properties of tellurium nanowires, they exhibit piezoelectric characteristics without the need for polarization processing. Furthermore, the silver nanowire electrode crafted through aerosol jet printing (AJP) displayed exceptional electrical conductivity and tensile properties, eliminating the requirement for sintering. Extrusion printing was used to print a silicone film as a stretchable substrate and electrical insulation between the printed tellurium and silver. The printed wearable piezoelectric device can be attached to a person's wrist to detect different gestures or to the neck to detect a heartbeat without using an external power source.

The literature reveals that AMSSID has gained widespread usage and significant advancements have been achieved in enhancing and applying numerous AM methods. Nevertheless, there persist challenges pertaining to the printing process, software, economic efficiency, and other pertinent aspects. We delve into these challenges in the subsequent sections.

The equipment used for the AMSSID is limited, and it is often difficult to fabricate SSIDs with complex structures and multiple materials. The most common processes for AMSSID include extrusion (FDM and DIW), Inkjet printing, and photocuring (SLA and DLP). However, other printing methods can be used for the AMSSIDs, such as metal printing. For example, SLM can be used to prepare metal components with strain self‐sensing functions by designing special structures. In addition, because SSIDs can have various sensing functions, multiple materials are required. Moreover, the materials have different characteristics, causing fabrication challenges using a single printing process. For example, the materials used in some SSIDs include insulating and conductive materials. The insulating materials can be printed using FDM, but DIW is required to fabricate conductive materials; thus, the two processes must be integrated. Finally, in contrast to traditional manufacturing processes that have undergone centuries of material development, the material development for additive manufacturing (AM) remains relatively immature. Although AMSSID can manufacture items using a wide variety of plastics and metals, the selection of available raw materials is not exhaustive because not all metals or plastics can be exposed to temperatures sufficiently high to conduct AM. More process development and research are required.

Multiple processes are often required in AMSSIDs, increasing software complexity. The primary software used in AMSSIDs includes design, 3D modeling, and print control software. Firstly, the design of SSIDs necessitates meticulous consideration of multiple scales and materials, posing stringent demands on the design software. It is imperative that the software possesses high accuracy to precisely model minute structures at the macro scale. Secondly, the 3D modeling of SSIDs is intricate due to the diverse scales and materials involved, leading to extended computation time and potential errors. A particularly formidable challenge lies in selecting appropriate printing parameters to yield accurate results. Lastly, the print control software must take into account various processes and facilitate seamless transitions between them, while also encompassing trajectory planning and control to attain the desired quality. For example, in the FDM/DIW approach, it is necessary to extrude part of the silk thread or use the printing plate to obtain better printing quality before FDM printing. When the process is switched to DIW, wire leakage or extrusion head wire leakage may occur. The print control software for AM equipment is highly complex, making software development difficult.

AM equipment is complex and often incorporates multiple processes. Therefore, it is a major challenge to improve the efficiency of AM equipment and reduce the printing cost of SSID devices. Many types of industrial AM equipment lag behind traditional mechanized equipment in terms of speed and efficiency, creating an obstacle to the wide use of AM devices. AM equipment has a more complex structure more complex processes, and higher quality requirements than traditional equipment. Therefore, more research and development investment is needed to improve the efficiency and reduce the cost of AMSSIDs. More importantly, current AMSSIDs lack unified functional and product quality standards, increasing the risk and cost of product development. It will be a long time before AMSSIDs are ready for commercial production and practical applications.

### 3.3 Printable Materials and Structures

Significant advancements have been achieved in the development of additive manufacturing (AM) materials, particularly in the realm of smart and functional materials. These materials are commonly employed for the fabrication of SSIDs, encompassing a range of materials such as conductive polymer composites, piezoelectric materials, and magnetic composites. It is crucial for these materials to fulfill specific functional requirements while exhibiting excellent printing performance.

**Figure 5:**
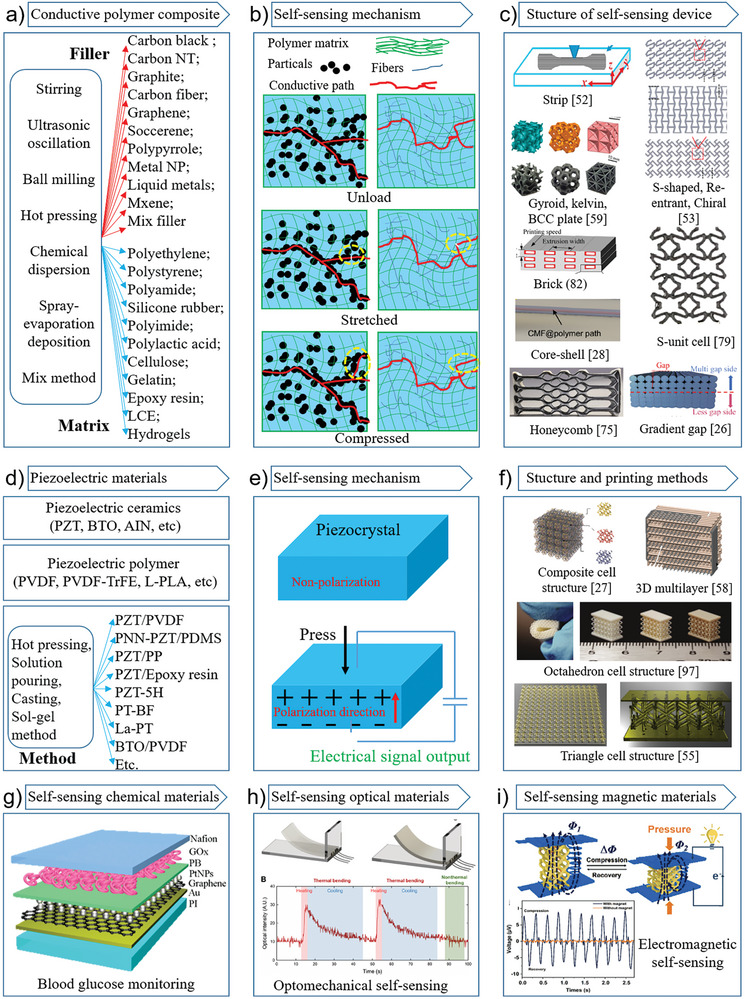
Currently printable materials for additive manufacturing self‐sensing intelligent device (AMSSID). a) Conductive polymer composite materials (CPCM) are currently the most common materials used for AMSSID printing. CPCM is mainly composed of different kinds of conductive fillers and polymer materials; b) the mechanism of CPCM self‐sensing is that under the action of strain, the number of conductive paths inside the composite material changes, so that the resistance changes. The strain variation of CPCM can be caused by mechanical action or by temperature; c) with different printing equipment, the CPCM material can be printed into different shape structures to achieve a variety of functions, or to improve its sensing performance, these shapes include, long strip,^[^ ^52^ ^]^ gyroid, kelvin, BCC plate,^[^ ^59^ ^]^ S‐shaped, re‐entrant, chiral,^[^ ^53^ ^]^ brick,^[^ ^82^ ^]^ S‐unit,^[^ ^79^ ^]^ core‐shell,^[^ ^28^ ^]^ honey comb,^[^ ^75^ ^]^ gradient gap,^[^ ^26^ ^]^ etc; d) piezoelectric material is an ideal AMSSID printing material, mainly including piezoelectric ceramic materials, piezoelectric polymer materials, and composite piezoelectric materials; e) the mechanism of piezoelectric materials is mainly that when subjected to external pressure, polarization is generated inside the material, which makes the charge on the upper and lower surfaces unbalanced, thus generating voltage; f) similarly, by printing different structures, piezoelectric materials can achieve more functions and perform better. These structures include, composite cell structure,^[^ ^27^ ^]^ 3D multilayer,^[^ ^58^ ^]^ octahedron cell structure,^[^ ^97^ ^]^ triangle cell structure,^[^ ^55^ ^]^ etc; g) AMSSID can be made by printing materials that can react specifically with certain chemicals, such as self‐sensing monitoring of glucose;^[^ ^39^ ^]^ h) sometimes it is also necessary to monitor its own optical characteristics, such as the monitoring of its own transparency,^[^ ^29^ ^]^ which can provide a good operating lens for imaging equipment and observation equipment. Self‐sensing materials with optical properties will play a huge role in many areas; i) materials with electromagnetic induction can sense their own magnetic^[^ ^98^ ^]^ signal changes, and can realize wireless self‐sensing. It will play an important role in implantable AMSSID. Reprinted with permission.^[^ ^28^, ^52^, ^53^, ^55^, ^58^, ^75^ ^]^ Copyright 2021, 2022, 2022, 2022, 2021, 2023. Elsevier; Reproduced with permission^[^ ^26^, ^59^, ^82^, ^98^ ^]^ Copyright 2022, 2022, 2020, 2020. Wiley; Reprinted with permission.^[^ ^79^ ^]^ Copyright 2022. American Chemical Society; Reproduced with permission^[^ ^27^, ^29^, ^39^, ^97^ ^]^ Copyright 2022, 2022, 2021, 2021. AAAS.

Conductive polymer composite materials (CPCMs) are well‐suited for AMSSID (**Figure** **5**a). They are commonly fabricated using a mixed method and consist of well‐insulating polymer material as the substrate and conductive nanoparticles as the filler. The substrate can be a polymer, including polyethylene, polypropylene, polystyrene, polyamide, silicone rubber, polyimide, polylactic acid, cellulose, gelatin, epoxy resin, and hydrogels. The fillers are generally carbon materials and metals. The carbon materials include carbon black, carbon nanotubes, carbon fibers, graphene, footballene, and graphite, and the metal materials include gold, silver, copper, aluminum, and other nanomaterials, as well as liquid metal materials. The base material and the filler are combined into a composite material, or various fillers and the base material can be integrated. For example, Verma et al.^[^ ^52^ ^]^ prepared multi‐wall carbon nanotubes (MWCNTs)/polypropylene random copolymer (PPR) composites and used FFF to print self‐sensing functional components. They used different proportions of MWCNTs (4, 6, or 8 wt%) and obtained the best temperature‐sensing performance when the mass fraction was 4%. The positive temperature coefficient was −12800 × 10^−6^/°C. The gauge factor was 395 when the temperature of the components was 60 ° C. Ye et al.^[^ ^82^ ^]^ prepared intelligent components with a strain‐sensing function using FFF. The self‐sensing component consisted of a continuous carbon fiber/ polylactic acid composite material. The intelligent component had high mechanical strength. The tensile strength of the component was 3.36 times higher, the flexural stress was 3.24 times higher, the tensile modulus was 5.1 times higher, and the flexural modulus was 4.9 times higher than those of polylactic acid. Furthermore, the tensile strain exhibited by the self‐sensing component was in close correspondence with its resistance, demonstrating excellent self‐sensing capabilities.

The conduction mechanism of composite conductive polymer materials is relatively complex (Figure 5b). Two theories have been proposed by scholars: the conduction path theory and electron tunnel theory of quantum mechanics. The conductive path theory states that the conductive particles in the system contact each other, and the electrons migrate through the conductive path to generate a current. The electron tunnel theory explains the conductivity of a system when conducting particles are adjacent but do not touch. CPCMs usually exhibit insulator‐conductor transition characteristics. A correlation exists between the resistance of composite materials and the quantity of conductive filler present. Upon reaching a specific threshold of filler content, the resistance of the composite materials experiences a marked exponential decline, a phenomenon commonly known as percolation.

In addition, composites often exhibit resistance‐temperature and resistance‐strain effects. The resistance‐temperature effect includes two aspects. A positive temperature effect occurs when the resistance of the system increases with the temperature, and the maximum mutation occurs at a specific temperature, the transition temperature. As the temperature gradually increases, the resistance decreases, and the material transitions from a conductor to a semiconductor or even an insulator. This phenomenon is commonly referred to as the negative temperature effect. Some materials exhibit a negative temperature effect followed by a positive temperature effect or only a negative temperature effect. The resistance‐strain effect is similar to the temperature effect, and positive and negative strain effects occur. The temperature and strain effects exhibited by conductive polymer composites are relatively complicated, and the principle differs for different composites. Research has revealed that the temperature and strain effects arise due to the disparities in the coefficients of thermal expansion between the base material and the filler. For example, we assume that the coefficient of thermal expansion is *σ*_1_ for the base material and *σ*_2_ for the filler. When the temperature changes, the coefficient of the filler changes with the temperature because *σ*_1_ ≠ *σ*_2_. When *σ*_1_ > *σ*_2_, the temperature increases due to the expansion of the base material, and the distance between the fillers changes. Within the composite, several paths become disconnected or the distance between them increases, leading to a reduction in the number of electronic channels or a weakening of the electronic tunneling effect. This increases the resistance, resulting in a positive temperature effect. In contrast, the resistance decreases when the temperature decreases. The temperature and strain effects of composite materials are exploited for creating self‐sensing devices. The temperature effect can be used to sense the temperature, and the strain effect is used to sense stress in the device.

Similarly, the strain effect observed in CPCMs is primarily attributed to variations in the distance between the conductive fillers within the composite material. When the shape of the material changes, the substrate generates strain, changing the distance between the particles and the resistance. Therefore, CPCMs are highly suitable for fabricating strain‐sensing devices. Since the resistance‐temperature and resistance‐strain effects of CPCM materials depend on the distance, it is difficult to distinguish the signals when both effects occur. Distinguishing between the two signals remains a major problem for CPCMs. In most cases, only a single signal is considered in SSIDs.^[^ ^26^ ^]^

The dominant advantage of AMSSIDs is the ability to create devices with various shapes and structures (Figure 5c), resulting in different functions and performances. A suitable combination of materials and structures can provide more and better self‐sensing functions. For example, Shao et al.^[^ ^51^ ^]^ prepared a photoresponsive liquid‐gas phase change elastomer (PRPTE) capable of sensing the deformation of graphene, microdroplets with a low boiling point, and silicone rubber. The PRPTE had excellent mechanical properties. The axial force generated by the liquid‐gas phase transition at a low boiling point was 400 times the materials’ weight at 100 °C, and the stability was high. Utilizing DIW, the team crafted flexible actuators capable of sensing and executing light‐controlled programmed movements, encompassing bending, grasping, and crawling actions. Furthermore, the capacitance‐based PRPTE exhibited autosensing functionality. Notably, the graphene component effectively absorbed near‐infrared light, converting it into heat. The liquid‐gas phase transition occurred at a low boiling point, and the dielectric constant decreased. The graphene was dispersed by the expansion of silicone rubber; the dielectric constant of the elastic body decreased, and the electrode spacing increased. As a result, the capacitance of the PRPTE decreased rapidly, enabling the real‐time perception of its deformation. The team created an artificial muscle that used a feedback mechanism to regulate muscle contraction and stretching to perform complex movements. The artificial muscle accurately sensed the leg bending angle through capacitance feedback, enabling precise control of its movements. This research integrated the driving/sensing functions of the flexible actuator, providing new insights into the design and manufacturing of soft robots with integrated self‐sensing abilities. Almahri et al.^[^ ^53^ ^]^ studied the effect of different printing structures on the performance of strain‐sensing components. An MWCNT/high‐density polyethylene (HDPE) composite material was meticulously prepared, and intricate S‐shaped and spiral self‐sensing components exhibiting a negative Poisson's ratio were skillfully printed utilizing FFF. The sensing coefficients were obtained for different shapes, and it was found that the S‐shaped structure had the optimum mechanical and self‐sensing properties. Inspired by the cellular structure of natural materials, Ubaid et al.^[^ ^59^ ^]^ developed a lightweight, 3D printable, and smart material using FFF to sense structural changes. The team successfully blended a widely used industrial plastic with carbon nanotubes, resulting in a material that surpasses its traditional counterpart in terms of toughness, strength, and intelligence. While plastic alone is non‐conductive, the composite material becomes electrically conductive due to the nanotubes carrying an electric charge. When the structure is subjected to mechanical loads, its resistance changes, which is called piezoresistivity. It enables materials to sense their structural health. In addition, the researchers used advanced 3D printing technology to create complex, mesoscale, porous structures with low weight and good mechanical properties. The design of the honeycomb material was inspired by light, strong, and porous materials found in nature, such as honeycombs, sponges, and bones. This material holds immense potential for practical applications across various fields. Researchers envision its utilization in medicine, prosthetics, as well as automotive and aerospace design, highlighting its diverse and promising applications. These fields require low‐density, tough materials with self‐sensing capabilities to fabricate lighter, more efficient structures or create back braces for people with scoliosis to sense if the patient's body is optimally supported. The material can also be used to make new battery electrodes. Verma et al.^[^ ^79^ ^]^ used FFF to fabricate intelligent components with strain self‐perception. A carbon nanotube/PPR composite was prepared. The strength and toughness of the composite were 40% higher, the toughness was 56% higher than that of the X material, and the electrical conductivity was 10^−4^ to 10^−1^ S m^−1^. The sensing factor k of the component was 10.1–17.4. Furthermore, self‐sensing components with auxetic re‐entrant and S‐unit cell lattices were printed. These materials exhibited tunable strain and damage sensitivity properties, making them suitable for use as orthopedic devices in biomedical engineering. For instance, self‐sensing orthopedic stents can provide valuable information that can be leveraged to enhance treatment outcomes and optimize stent design.

Piezoelectric materials produce a charge on the surface when exposed to mechanical stress, called the piezoelectric effect (Figure 5d). When the charge value is proportional to the mechanical stress, it is called the positive piezoelectric effect (Figure 5e). In contrast, when the piezoelectric material produces a geometric strain of equal proportion when exposed to an electric field, it is called the inverse piezoelectric effect. Since the discovery of the piezoelectric effect in 1880, piezoelectric materials have undergone extensive development, encompassing quartz crystals, polycrystalline materials, polymers, ceramics/polymer composites, single‐crystal materials, and numerous other substances. While piezoelectric ceramics possess desirable piezoelectric properties, they fall short in terms of physical characteristics and exhibit low ductility, thus limiting their application in flexible sensors and actuators. With the rapid development of science and technology, flexible integrated circuits, wearable electronic devices, and flexible intelligent robots have been developed for use in daily life and industry. Hard and brittle ceramic materials are unsuitable. In addition, the processing level of piezoelectric ceramics is high, and it is difficult to fabricate them using existing AM methods. Thus, piezoelectric polymers and piezoceramic/polymer composites are currently the most widely used materials for the AMSSIDs.^[^ ^54^, ^55^, ^56^, ^57^ ^]^ Similarly, with the help of additive manufacturing technology, piezoelectric materials can better play the self‐sensing function. First, additive manufacturing is theoretically capable of creating any shape, making these piezoelectric materials a better match for smart devices. More importantly, additive manufacturing can endow piezoelectric materials with more microstructures, some of which can enhance piezoelectric properties, some can enhance mechanical properties, and some can realize piezoelectric anisotropy, so that more functions and better performance can be achieved (Figure 5f). These materials can be conductive materials that act as electrodes for the piezoelectric materials, or insulating materials that protect the piezoelectric materials and prevent leakage.

Polyvinylidene difluoride (PVDF) is the most commonly used material in the AM of piezoelectric polymeric materials. A PVDF polymer has *α*, *β*, *γ*, and *δ* phases. After melting and recrystallization, the α phase emerges. Within this phase, dipole motions mutually neutralize, rendering it non‐polar. Subsequently, all orthogonal‐trans α phases undergo a transformation into the all‐trans β phase. This β phase exhibits an all‐trans planar zigzag configuration, characterized by a parallel alignment of dipole moments within the cell. This arrangement allows for the integration of various polarities. Thus, the *β* phase has the highest piezoelectric effect among all PVDF crystal forms. It is worth noting that stretching must be followed by annealing because PVDF undergoes lattice distortion due to mechanical stretching. The copolymer vinylidene fluoride and polyvinyl chloride trifluoride (TrFE) yields a stable polarized random *β*‐phase copolymer (PVDF‐TrFE). The polymer does not require special treatment but can be directly polarized to obtain piezoelectric properties; however, it is relatively expensive. For example, Yuan et al.^[^ ^56^ ^]^ successfully printed a multilayer PVDF‐TrFE piezoelectric film using solution‐assisted 3D printing and designed a rugby ball‐type piezoelectric energy harvester (PEH). The 6‐layer PVDF‐TrFE film had the largest piezoelectric coefficient (d33 ≈ 130 pC N^−1^). Due to the force amplification properties of the football shape and the piezoelectric effect inherent in the multilayer composite film, the PEH achieved a peak output voltage of 88.6 Vpp under a pressure of 0.046 MPa in the low‐frequency range. This performance is remarkable, exceeding that of a PEH constructed from a single‐layer planar film by a factor of two.Its peak output power density (16.41mW cm^−2^) was almost 22 times that of the planar PEH. The PEH had a unique bending mechanism with efficient mechanical conversion. This study provided inspiration for 3D‐printed piezoelectric sensors but did not fully reflect the 3D design freedom of 3D printing. Bernasconi et al.^[^ ^99^ ^]^ proposed an intelligent combination of 3D printing and inkjet material deposition. The structural components of accelerometers were created using stereolithography and photocured resins, whereas the electrodes for the PVDF‐TrFE piezoelectric layers and the silver for acceleration readings were fabricated by 3D inkjet printing. The results showed that the proposed hybrid AM technique was promising to manufacture mesoscale electromechanical sensors.

Apart from PVDF and its copolymers, numerous other piezoelectric polymers have been discovered and extensively utilized, including PLA, a prominent biomass‐derived polymer. The lactic acid monomer possesses asymmetric carbon atoms, and when L‐lactide undergoes polymerization, it forms an L‐type PLA or poly(L‐lactide acid) (PLLA). These polymers exhibit shear piezoelectric properties when subjected to stretching or elongation.

Piezoelectric composite material refers to the combination of two or more materials derived from physical or chemical methods. The composite material can have physical or chemical properties that single materials do not have. Piezoelectric ceramics indeed exhibit a high piezoelectric effect and a large electromechanical coupling coefficient, however, their inherent hardness and brittleness pose significant limitations on their further development. The discovery of piezoelectric polymers has resulted in the development of flexible piezoelectric materials and devices. The piezoelectric effect of piezoelectric polymers is less pronounced than that of piezoelectric ceramics. Piezoelectric composites consisting of piezoelectric ceramics and polymers have a good development prospect because of their excellent plasticity. Piezoelectric composites possess a superior piezoelectric effect compared to piezoelectric polymers, while also exhibiting greater flexibility and ductility than piezoelectric ceramics. Consequently, they have garnered significant attention from researchers worldwide. Two factors affect the performance of piezoelectric composites. One is the performance of the components of the piezoelectric composites, and the other is the structural design of the piezoelectric composites. The effects of composite materials can be categorized into three distinct types: additive, combined, and product effects. Each of these effects plays a crucial role in enhancing the properties of composite materials. The high plasticity of piezoelectric composites enables their use in AM to fabricate efficient piezoelectric devices. For example, Zhang et al.^[^ ^54^ ^]^ used surface projection micro‐stereolithography to design and adjust the interface compatibility of piezoelectric boron nitride nanotubes (BNNTs) and photosensitive polymer resin and optimize the topology. A flexible piezoelectric sensor with high sensitivity, wide response, and retractable structure was fabricated. In another instance, the low interfacial adhesion between the polymer matrix and the ceramic powder results in inefficient stress transfer, which subsequently degrades the piezoelectric properties of incompatible, low‐dispersive printed composites. In addition, it is difficult to obtain good printing adaptability and piezoelectric response at the same time because higher ceramic concentrations lead to high scattering, agglomeration, and high viscosity. Ceramic‐polymer composites must have a high ceramic content, suitable dispersibility, and good interfacial adhesion to 3D print composites with good piezoelectric properties. Chang et al.^[^ ^55^ ^]^ used DLP to fabricate thick piezo‐polymer composites. The 3D‐printed piezoelectric composite, which comprised a functionalized piezoelectric ceramic powder and dispersant, exhibited remarkable flexibility, a noteworthy high voltage coefficient (dss), and a piezoelectric voltage output approximately three times greater than that of conventional planar structures. The fabricated sensor had a high open‐circuit voltage and can be used as a wearable and stretchable self‐powered device, a high‐performance tactile position sensor, and a self‐sensing device for energy harvesting in pressure monitoring systems. Furthermore, Liu et al.^[^ ^97^ ^]^ used 3D printing of a micro‐scale continuous liquid interface to manufacture piezoelectric metaprametics. They performed chemical surface‐modification of nanoscale particles using commonly used piezo‐ceramic materials, such as barium titanate (BTO), and conducted light‐curing of 3D printed resin containing up to 30 wt% of ceramic nanoparticles. The possibility of applying this technology to other common piezoelectric ceramic materials, such as lead zirconate titanate (PZT) and aluminum nitride (AlN), was verified, demonstrating the wide application range of this technology. Finally, Piezoelectric metamaterials with diverse structures were fabricated, printed, and thoroughly characterized to confirm the viability of utilizing this technology in the creation of 3D piezoelectric metamaterials for flexible electronics, wearable devices, and various other applications. Yan et al.^[^ ^58^ ^]^ configured polydimethylsiloxane (PDMS) and PNN‐PZT ceramics into a slurry suitable for 3D printing in a certain proportion. A flexible piezoelectric sensor similar to a multilayer ceramic capacitor was successfully fabricated. In the era of rapid development of artificial intelligence, this flexible multi‐layer structure has great potential for use as electronic skin or artificial muscles.

In addition to conductive polymer composites and piezoelectric materials, there are many materials to realize a variety of different self‐sensing functions, including self‐sensing chemical materials, self‐sensing optical materials, and self‐sensing magnetic materials. However, there are few studies on these materials, and only a few relevant reports in the literature. For example, Pu et al.^[^ ^39^ ^]^ use materials that can interact with glucose for self‐sensing detection of blood glucose (Figure 5g). Li et al.^[^ ^29^ ^]^ utilize optical materials from perceived transparency (Figure 5h). Wu et al.^[^ ^98^ ^]^ used magnetic material printing to realize the conversion of pressure and electrical signals (Figure 5i). These materials will be particularly important in future self‐sensing applications. Because these materials extend the field of sensing from mechanical quantities to chemical and electromagnetic quantities. This expansion will make the self‐sensing signal more abundant, and will also make AMSSID realize more functions, so as to create better intelligent products. However, it is not easy to print and prepare these materials, and it is still a great challenge compared with current printing equipment. With the advancement of printing equipment and technology, these challenges will also be solved.

In summary, much progress has been made in the development of self‐sensing materials, although continuous improvement and development are required. The precise control of the conductivity and sensing properties of conductive polymer composites remains a significant challenge. The conductivity, mechanical properties, and sensing capabilities of composite materials are significantly influenced by the intricate relationship between the filler and polymer materials, as well as the filler's content, distribution, and size within the polymer matrix. The accurate control of the filler in the polymer matrix will improve electrical conductivity, mechanical properties, and sensing properties. Piezoelectric materials have an advantage as self‐sensing materials due to their piezoelectric characteristics; they have broad application prospects in future self‐sensing devices. However, the fabrication of self‐sensing functional devices using piezoelectric materials must overcome several problems, including low piezoelectric energy efficiency, processing difficulties, and additional electrodes. Furthermore, there have been limited studies exploring other types of self‐sensing materials, including electromagnetic, optical, and chemical self‐sensing materials. The advancement of these materials holds the potential to significantly enhance the performance of self‐sensing functional devices.

## 4 Current Challenges and Future Development

AMSSIDs is still evolving from a concept to a practical methodology that enables the fabrication of intelligent, multi‐functional, and self‐sensing components. Thanks to the constant advancements in science and technology, as well as the interdisciplinary nature of additive manufacturing, numerous opportunities arise to further enhance this method through scientific discoveries, technological breakthroughs, and industrial applications. We highlight several trends and development frontiers.

AMSSID has the capability to process suitable materials into appropriate structures, and configure them in precise positions, enabling them to perform diverse self‐sensing functions. However, more self‐sensing functions are required. The most significant advantage of SSIDs is the monitoring of the device. Different self‐sensing functions can be created by monitoring different device signals. Most SSIDs focus on monitoring their mechanical signals, including stress, strain, and structural damage. However, monitoring other signals represents a challenge, especially chemical and optical signals. Contrary to mechanical signals, chemical signals rely on the interactions between chemical substances to accomplish material transformation. This transformation is converted into detectable signals, although their detection remains challenging. There are limitations in the self‐sensing function of optical signals due to optical materials and their structure. Fabricating SSIDs capable of sensing optical signals using additive manufacturing is challenging due to the micro and nano‐level wavelengths of visible light. Most AM equipment lacks the ability to process micro and nanostructures, thus limiting their application in this domain.

The preparation of AMSSID requires further improvement. Various sensors and actuators with different functions at the material level can be integrated, but the design of AMSSIDs with multi‐sensing functions is relatively complex because multiple, heterogeneous, and multi‐functional materials and several scales are involved. In addition, existing functional materials and AM manufacturing equipment must be used. These requirements result in challenges in designing software and algorithms. Establishing a standardized design methodology and criteria for AMSSID will facilitate the development of design software tools and pave the way for the creation of numerous innovative AM methods tailored for SSIDs. A lack of unity and standards exists in the performance evaluation of AMSSIDs. Typically, the device's performance is evaluated, but a lack of methods and criteria exist to perform a consistent evaluation of AMSSIDs.

The existing AM equipment frequently falls short of meeting the requirements necessary to fabricate AMSSIDs with intricate structures. Thus, it is necessary to carry out secondary development or improve the existing equipment to meet the requirements. Therefore, further development of AM equipment is required. Due to the limitations of the materials that can be printed by the current equipment, it is imperative to develop additional processing and printing techniques. In addition, SSIDs have micro‐ and nano‐scale structures and require high structural accuracy, making it necessary to improve printing accuracy and efficiency. SSIDs often consist of multiple materials at different scales. Hence, as additive manufacturing, multi‐process integration, or hybrid processes become more widespread, there will be a need for more sophisticated hardware designs and software systems. The integration of multiple processes is a significant challenge in the development of AM equipment.

Most materials utilized for AMSSIDs necessitate specific functionalities, yet the commercial market currently offers a scant selection of such materials. Consequently, the range of materials suitable for AMSSID is significantly limited. Thus, more functional materials or new materials with more complex processes are urgently needed for AMSSIDs. Additionally, improving the stability and the structural design of conductive composites and distinguishing different signals (temperature and strain) in the resistance signal of the composite are the main challenges. The key challenges lie in enhancing the piezoelectric properties of the materials post‐printing, configuring electrode materials appropriately for seamless printing, and achieving multifunctional electromechanical conversion. In addition, self‐sensing functional materials require further development, including magnetic, optical, and self‐healing functional materials.

## Conflict of Interest

The authors declare no conflict of interest.
